# Predictive roles of serum IL-33/ST2 and BDNF in depressed patients with repetitive transcranial magnetic stimulation: a pilot study

**DOI:** 10.3389/fnhum.2026.1828777

**Published:** 2026-06-01

**Authors:** Shuang Wu, Xiaofeng Zuo, Ling Liu, Mengshu Yang, Xin Li, Shuiying Li

**Affiliations:** 1 Mental Health Center, National Center for Mental Disorders, West China Hospital, Sichuan University, Chengdu, China; 2 West China School of Nursing, Sichuan University, Chengdu, Sichuan, China

**Keywords:** BDNF, biomarker, depression, IL-33, repetitive transcranial magnetic stimulation

## Abstract

**Background:**

Major depressive disorder (MDD) is considered a serious public health issue and adversely affects individuals’ quality of life. Repetitive transcranial magnetic stimulation (rTMS) is a crucial strategy for treating MDD. This pilot study aims to investigate the relationship between serum IL-33/ST2 and BDNF levels and treatment improvement in MDD patients receiving rTMS therapy.

**Methods:**

This prospective study enrolled 36 patients diagnosed with MDD (under the DSM-5 diagnostic criteria) between July 2023 and July 2024. All patients underwent standard 10 Hz high-frequency rTMS for the left dorsolateral prefrontal cortex (DLPFC) for 4 weeks (totaling 20 sessions). Fasting venous blood was collected before treatment and at the end of treatment. Serum levels of IL-33, sST2, 5-HT, IL-6, and BDNF were measured using ELISA. Clinical efficacy was assessed using the Hamilton Depression Rating Scale-17 items (HAMD-17), with a reduction rate ≥50% defined as treatment response. Statistical analyses included correlation analysis, intergroup comparisons, and ROC curves, and analyses of pre-to-post-biomarker changes (*Δ*) in relation to both response status and symptom reduction.

**Results:**

A total of 36 MDD patients received rTMS treatment for 4 weeks. Among them, 20 patients (55.6%) responded significantly. At baseline, serum IL-33 levels were positively correlated with HAMD-17 scores (r = 0.41, FDR-adjusted *q =* 0.02), whereas sST2 levels were negatively correlated (r = −0.68, FDR-adjusted *q <* 0.001). After rTMS treatment, those responders exhibited significantly lower serum IL-33 levels (FDR-adjusted *q =* 0.007) but higher BDNF levels (FDR-adjusted *q =* 0.005) compared to those non-responders. Those responders also had significantly greater increases in BDNF (ΔBDNF, FDR-adjusted *q =* 0.0004) and sST2 (ΔsST2, FDR-adjusted *q =* 0.021) compared to non-responders. ΔBDNF levels (*ρ* = 0.499, FDR-adjusted *q =* 0.0049) were positively while ΔIL-33 levels (r = −0.477, FDR-adjusted *q =* 0.0054) were negatively correlated with symptom improvement. ROC analysis revealed that baseline 5-HT levels could predict treatment response (AUC = 0.834, *p <* 0.001), and post-treatment IL-33 (AUC = 0.916, *p <* 0.001) and BDNF (AUC = 0.916, *p <* 0.001) levels were significantly associated with rTMS treatment response.

**Conclusion:**

Effective rTMS treatment in MDD patients is associated with decreased serum IL-33 levels and increased BDNF levels. The IL-33/ST2 and BDNF pathways may be involved in the antidepressant mechanism of rTMS.

## 1 Introduction

From 1990 to 2019, the global number of Disability-Adjusted Life Years(DALYs) caused by mental disorders increased from 80.8 million (95% uncertainty interval [UI] 59.5–105.9) to 125.3 million (93.0–163.2), and the proportion of global disability-adjusted life years attributable to mental disorders rose from 3.1% (95% UI 2.4–3.9) to 4.9% (3.9–6.1), with depression ranking first among all mental disorders (GBD 2019 Mental Disorders Collaborators, 2022). Major Depressive Disorder (MDD) is a highly prevalent and disabling major mental illness, and its core pathophysiological mechanisms remain incompletely elucidated. MDD is characterized by at least one discrete depressive episode lasting at least 2 weeks, involving significant changes in mood, interest, cognition, and vegetative symptoms (Otte et al., 2016). MDD is one of the leading contributors to the global disease burden, with its incidence increasing by 26.60% from 1990 to 2019 (Hong et al., 2022). In 2020, the global DALYs for MDD were 49.4 million (COVID-19 Mental Disorders Collaborators, 2021). In 2021, the incidence, prevalence, and DALYs of MDD in China increased significantly with age, with the highest incidence observed in the 70 + age group. Additionally, females are more susceptible to MDD than males (Wang et al., 2025). MDD is a heterogeneous, complex, and multidimensional disease, and its etiology has not been fully elucidated, with biological, psychological, and psychosocial factors playing significant and intertwined roles. Most patients with depressive disorders exhibit significant social functional impairment (Jauhar et al., 2023). MDD ranks 13th among all diseases in terms of global disease burden and 11th in China (Lu et al., 2021). The ultimate goal of MDD patient management is functional recovery, which can be achieved through personalized, comprehensive, and recovery-oriented interventions (Sampogna et al., 2024). Currently, pharmacology and neuromodulation are mainstream treatments for MDD, but they still have certain limitations, including treatment side effects and tolerability. In practical applications, interventions should be selected based on patients’ needs and preferences to tailor treatment plans through shared decision-making approaches (Njenga et al., 2024).

Previous studies have confirmed that mood disorders are associated with an imbalance in the function of the dorsolateral prefrontal cortex (DLPFC). Depressed patients typically exhibit abnormally reduced left DLPFC function and abnormally enhanced right DLPFC function (Fitzgerald et al., 2009). Repetitive transcranial magnetic stimulation (rTMS) stimulates cortical neurons in the brain through electromagnetic pulses. In the mid-1990s, the antidepressant effects of rTMS were first confirmed, and it has been approved by the U. S. Food and Drug Administration (FDA) for the treatment of various neuropsychiatric disorders, including obsessive-compulsive disorder, smoking cessation, migraines, and depression (Perera et al., 2016). For depression, rTMS is used to treat patients who do not respond to medications, with response and remission rates of 50 and 20%, respectively (Gogulski et al., 2023). Although many rTMS parameters can be adjusted, including brain targets, patterns, frequency, intensity, and pulse number, only a few parameters are commonly used clinically: 10 Hz and theta-burst stimulation (TBS) targeting the left dlPFC and 1 Hz targeting the right dlPFC. Therefore, there remains a lack of ability to stratify patients by predicting responses or to optimize rTMS through personalized treatment parameters. This limited utilization represents a missed opportunity, as a better understanding of how each parameter affects clinical outcomes would enable personalization (Gogulski et al., 2023; Hopman et al., 2021).

Depression has been considered a disease involving excessive activation or suppression of the immune system. Markers of impaired cellular immunity (reduced natural killer cell cytotoxicity) and inflammation (elevated IL-6, TNF-*α*, and CRP) are associated with depression (Blume et al., 2011). Interleukin-33 (IL-33), a multifunctional cytokine belonging to the IL-1 family, is expressed in various cell types, including microglia, astrocytes, epithelial cells, and endothelial cells. Meanwhile, its receptor ST2 is highly expressed in microglia and astrocytes (He et al., 2022). A study analyzing the human brain database of IL-33 in the Allen Human Brain Atlas (AHBA) revealed that IL-33 exhibits higher differential expression in brain regions critical for emotional functions (such as the paraventricular nucleus of the hypothalamus, amygdala, and cingulate gyrus) compared to other commonly studied cytokines like IL-6 and IL-1β (Wang et al., 2021). As an “alarmin” cytokine, IL-33 is released during tissue or cellular injury and activates downstream pathways upon binding to its receptor ST2, altering the expression of pro-inflammatory cytokines, chemokines, and Th2-related cytokines (Schiering et al., 2014). A recent study showed that compared to healthy controls (*n =* 125), MDD patients (*n =* 129) had reduced mean serum IL-33 levels (159.12 ± 6.07 pg./mL vs. 180.60 ± 8.64 pg./mL, *p* = 0.042) (Nahar et al., 2024). A meta-analysis indicates that IL-33 and ST2 levels in cerebrospinal fluid and serum are positively correlated with a reduced risk of MDD and bipolar disorder (BD). This suggests that changes in IL-33 levels in serum and cerebrospinal fluid could serve as useful indicators for assessing depression risk, and this biomarker offers potential therapeutic strategies to alleviate disease burden (Liu et al., 2023).

In addition to the immune-inflammatory pathway, the serotonin (5-HT) system and brain-derived neurotrophic factor (BDNF) represent two key molecules in the pathophysiology of MDD. 5-HT is one of the most important monoamine neurotransmitters in the central nervous system, and deficient 5-HT function lies at the core of the monoamine hypothesis of depression (Li, 2020). Currently available antidepressants such as paroxetine, sertraline, and fluoxetine are selective serotonin reuptake inhibitors (SSRIs) that elevate 5-HT concentrations in the brain by blocking its reuptake, thereby ameliorating low mood and loss of interest (Singh et al., 2025; Vijayakumar, 2025). BDNF, a critical mediator of neuroplasticity, underpins the neurotrophic hypothesis of depression; reduced BDNF levels have been associated with atrophy of the hippocampus and other brain regions in patients with depression (Arosio et al., 2021; Esalatmanesh et al., 2023). Previous meta-analyses have shown that serum BDNF is lower in depressed patients and increases significantly following treatment (Brunoni et al., 2008; Molendijk et al., 2014). A Korean clinical study enrolling 225 MDD patients and 1,469 healthy controls reported that the rs11030101 (BDNF) gene polymorphism was strongly associated with suicidal behaviours (Kim et al., 2025). Taken together, these lines of evidence indicate that 5-HT and BDNF play important roles in the pathophysiology of depression and the response to antidepressant treatment.

Based on the above research findings, we hypothesized that the levels of inflammatory cytokines (including IL-33 and IL-6) in the serum of MDD patients are correlated with disease severity and treatment responsiveness to rTMS. To test this hypothesis, we collected serum samples from MDD patients (*n =* 36) before and after rTMS treatment, measured the levels of 5-HT, IL-6, IL-33, soluble ST2 (sST2), and BDNF using ELISA, and examined the correlations between these biomarkers and depression scores (HAMD-17). We anticipate that this exploratory study will provide preliminary references for the personalized application of rTMS.

## 2 Materials and methods

### 2.1 Study design and ethical approval

This study consecutively included MDD patients who met the criteria and were treated at West China Hospital of Sichuan University from July 2023 to July 2024. This study strictly adhered to the principles of the Declaration of Helsinki and was approved by the Ethics Review Committee of West China Hospital of Sichuan University (no. 2023011). All enrolled patients were fully informed of the study content prior to participation and signed written informed consent forms.

### 2.2 Patient selection

*Inclusion criteria*: Age 18–65 years, regardless of gender. Meet the diagnostic criteria for MDD in the Diagnostic and Statistical Manual of Mental Disorders, Fifth Edition (DSM-5) (Tabuteau et al., 2022), confirmed by clinical interviews conducted by at least two attending psychiatrists. All patients received at least two antidepressant drug treatments, but did not meet the clinical efficacy criteria, thus meeting the diagnostic criteria for treatment-resistant depression (TRD). Participants were patients with treatment-resistant MDD for at least 12 months. During the rTMS treatment period, patients continued to receive the antidepressant treatment they had previously initiated in accordance with international guidelines, ensuring that the antidepressant regimen remained stable for at least 4 weeks before enrollment, or agreed to keep the regimen unchanged during the study period (including SSRIs, SNRIs, tricyclic antidepressants, etc.). Possess basic comprehension and communication skills, and be capable of cooperating to complete scale assessment and treatment.

*Exclusion criteria*: Diagnosis of bipolar disorder, schizophrenia spectrum disorders, or other primary psychiatric disorders. Presence of severe or unstable physical illnesses (e.g., heart, liver, or kidney failure; active infections; autoimmune diseases; malignancies, etc.). History of epilepsy, organic brain diseases, or contraindications to rTMS treatment such as intracranial metal implants. Pregnant or breastfeeding women.

### 2.3 rTMS treatment protocol

All patients received standard high-frequency rTMS treatment. According to the published literature (Hussain et al., 2024; Blumberger et al., 2018), the treatment plan was once a day, 5 times a week (from Monday to Friday), for a total of 4 weeks, amounting to 20 sessions. Specifically, the treatment was administered using a pulsed magnetic field stimulator (RT-100, JJWG-med). The stimulation target area was the DLPFC. During the localization process, the international 10–20 EEG system was employed, with the center of the coil positioned 5–6 cm anterior and lateral to the F3 electrode point. The stimulation frequency was 10 Hz, with each sequence lasting 4 s and intervals of 26 s between sequences, totaling 3,000 pulses per session. The stimulation intensity was set to 120% of the patient’s resting motor threshold (RMT). RMT was determined as the minimum output intensity required to elicit a motor evoked potential (MEP) of ≥50 μV in the contralateral first dorsal interosseous muscle of the hand. Treatment was administered 5 times per week (Monday to Friday) for 4 consecutive weeks, totaling 20 sessions. To ensure tolerability and safety, standardized procedures were followed for participants experiencing discomfort during stimulation, including minor adjustments to coil positioning or gradual reduction of stimulation intensity.

### 2.4 Clinical and laboratory assessment

Assessments using the HAMD-17 scale were conducted by psychiatrists who underwent uniform training and were blinded to patient grouping at two time points: before treatment and at the end of treatment (week 4) (Hamilton, 1960).

Calculate the HAMD-17 reduction rate before and after treatment: Reduction rate = (Baseline total score − Post-treatment total score) / Baseline total score × 100%. A HAMD-17 reduction rate ≥ 50% at the end of treatment is defined as treatment response, and a HAMD-17 total score ≤ 7 is defined as clinical remission or recovery (Geng et al., 2024).

Venous blood samples were collected at the two clinical assessment time points. All blood sampling was conducted in the morning before the first meal of all patients. Vacuum blood collection tubes without anticoagulants were used to collect approximately 5 mL of peripheral venous blood. After collection, the blood was left to stand at room temperature for 30 min and then allowed to naturally clot. Then, it was centrifuged at 3000 rpm for 15 min at 4 °C to separate the serum, which was aliquoted into pyrogen-free Eppendorf tubes and immediately stored at −80 °C in an ultra-low temperature refrigerator to avoid repeated freezing and thawing.

ELISA was used to quantitatively detect IL-33, sST2, IL-6, 5-HT, and BDNF in the collected samples. The following kits were employed: IL-33 (Beyotime, PI631), sST2 (Beyotime, PS848), IL-6 (Beyotime, PI325), 5-HT (Beyotime, U96-1002E-48 T), and BDNF (Beyotime, PB070). All procedures strictly followed the manufacturer’s instructions. According to the instructions of the test kit, the intra-assay coefficient of variation (CV) and inter-assay coefficient of variation (CV) for each indicator are both less than 10%. All samples were tested in duplicate, and the average values were taken. The laboratory personnel were blinded to the patients’ clinical groupings and treatment conditions.

### 2.5 Statistical analysis

All data analyses were performed using SPSS Statistics (version 21.0; IBM Corp., Armonk, NY, USA) software. Continuous variables are expressed as mean ± SD or median (interquartile range, IQR), and categorical variables are presented as frequency (%). The Shapiro–Wilk test was used to assess the normality of continuous variables, and variables that met the normal distribution were compared between the two groups using the independent sample *t*-test, while non-normal variables were compared using the Mann–Whitney U test. Categorical variables were expressed as frequencies (percentages), and the chi-square test or Fisher’s exact test (when the expected frequency < 5) was used for comparisons between groups. Correlations between variables were assessed using Pearson correlation analysis (for normally distributed variables) or Spearman rank correlation analysis (for non-normally distributed variables). ROC curve analysis was employed to evaluate the predictive performance of each indicator for treatment response, and the AUC with 95% CI was calculated. All statistical tests were two-sided, and a *p <* 0.05 was considered statistically significant. Since this study involved multiple biomarkers and multiple statistical tests (including correlation analysis, inter-group comparisons), we employed the Benjamini-Hochberg false discovery rate (FDR) method to adjust the *p*-values obtained from the correlation analysis and inter-group comparisons. Where applicable, the adjusted *p*-values (i.e., *q*-values) were also reported. After correction, a *q*-value less than 0.05 was considered statistically significant.

## 3 Results

### 3.1 Demographic characteristics of enrollled patients

This study included 36 MDD patients. A total of 17 cases (47.2%) were receiving SSRI treatment, 12 cases (33.3%) were receiving SNRI treatment, 5 cases (13.9%) were receiving TCAs treatment, and 2 cases (5.6%) were receiving other antidepressants (such as mirtazapine, bupropion) as monotherapy or combination therapy. Based on the HAMD-17 score reduction rate before and after 4 weeks of rTMS treatment, patients with a reduction rate ≥50% were classified as responders (Geng et al., 2024). Among them, 20 patients (55.6%) were categorized as responders, and 16 (44.4%) as non-responders. Basic patient information is shown in Table 1. In the responder group, there were 10 males and 10 females, with males accounting for 50.0%. The average age of patients was 28.05 ± 5.27. In the non-responder group, there were 9 males and 7 females, with males accounting for 56.3%. The average age of patients was 30.88 ± 5.28. Patients with hypertension and diabetes as underlying conditions numbered 2 each, accounting for 5.6%. All enrolled patients exhibited moderate to severe depressive symptoms at baseline. The median baseline HAMD-17 score for the entire cohort was 29.5 (9), with 77.8% (28/36) of patients having HAMD-17 scores ≥23, classified as Very Severe. As shown in Table 1, there were no significant differences between responders and non-responders in demographic and clinical characteristics, including age, sex, body mass index (BMI), marital status, education level, comorbidities (hypertension and diabetes), lifestyle habits (smoking and alcohol consumption), baseline HAMD-17 scores, and baseline depression severity classification (all *p* > 0.05).

**Table 1:** Baseline characteristics of patients with major depressive disorder receiving rTMS treatment.

Characteristic	Total (*n =* 36)	Responders (*n =* 20)	Non-responders (*n =* 16)	Test statistic	*p*-value
Age, mean ± SD	29.31 ± 5.39	28.05 ± 5.27	30.88 ± 5.28	t(34) = −1.60	0.119
Male, *n* (%)	19 (52.8)	10 (50.0)	9 (56.3)	*χ*^2^(1) = 0.14	0.709
BMI, mean ± SD	23.76 ± 4.07	23.40 ± 3.98	24.20 ± 4.28	t(34) = −0.58	0.566
Antidepressant use, *n* (%)				*χ*^2^(3) = 0.38	0.947
SSRIs	17 (47.2)	10 (50.0)	7 (43.8)		
SNRIs	12 (33.3)	6 (30.0)	6 (37.5)		
TCAs	5 (13.9)	3 (15.0)	2 (12.5)		
Others	2 (5.6)	1 (5.0)	1 (6.3)		
Marital status, *n* (%)				*χ*^2^(1) = 1.63	0.202
Unmarried	20 (55.6)	13 (65.0)	7 (43.8)		
Married	16 (44.4)	7 (35.0)	9 (56.3)		
Hypertension, *n* (%)	2 (5.6)	0 (0.0)	2 (5.6)	–	0.190
Diabetes mellitus, *n* (%)	2 (5.6)	0 (0.0)	2 (5.6)	–	0.190
Education, *n* (%)				*χ*^2^(2) = 1.42	0.491
junior high school and below	5 (13.9)	3 (15.0)	2 (12.5)		
high school	19(52.8)	12 (60.0)	7 (43.8)		
higher education	12 (33.3)	5 (25.0)	7 (43.8)		
Alcohol consumers, *n* (%)	12 (33.3)	8 (40.0)	4 (25.0)	*χ*^2^(1) = 0.90	0.343
Smoke, *n* (%)	15 (41.7)	9 (45.0)	6 (37.5)	*χ*^2^(1) = 0.21	0.650
HAMD-17 score, median(IQR)	29.50 (9)	30.00 (6)	28.50 (13)	U = 143	0.577
Depression level, *n* (%)				*χ*^2^(2) = 0.13	0.938
Moderate (14–18)	4 (11.1)	2 (10.0)	2 (12.5)		
Severe (19–22)	4 (11.1)	2 (10.0)	2 (12.5)		
Very Severe (≥23)	28 (77.8)	16 (80.0)	12 (75.0)		

Data are presented as Mean ± SD, Median (IQR), or *n* (%). rTMS, Repetitive Transcranial Magnetic Stimulation; HAMD-17, 17-item Hamilton Depression Rating Scale; BMI, body mass index; IQR, interquartile range; SSRIs, selective serotonin reuptake inhibitors; SNRIs, serotonin-norepinephrine reuptake inhibitors; TCAs, tricyclic antidepressants.

### 3.2 Correlation between baseline biomarker levels and baseline depression severity

**Figure 1:**
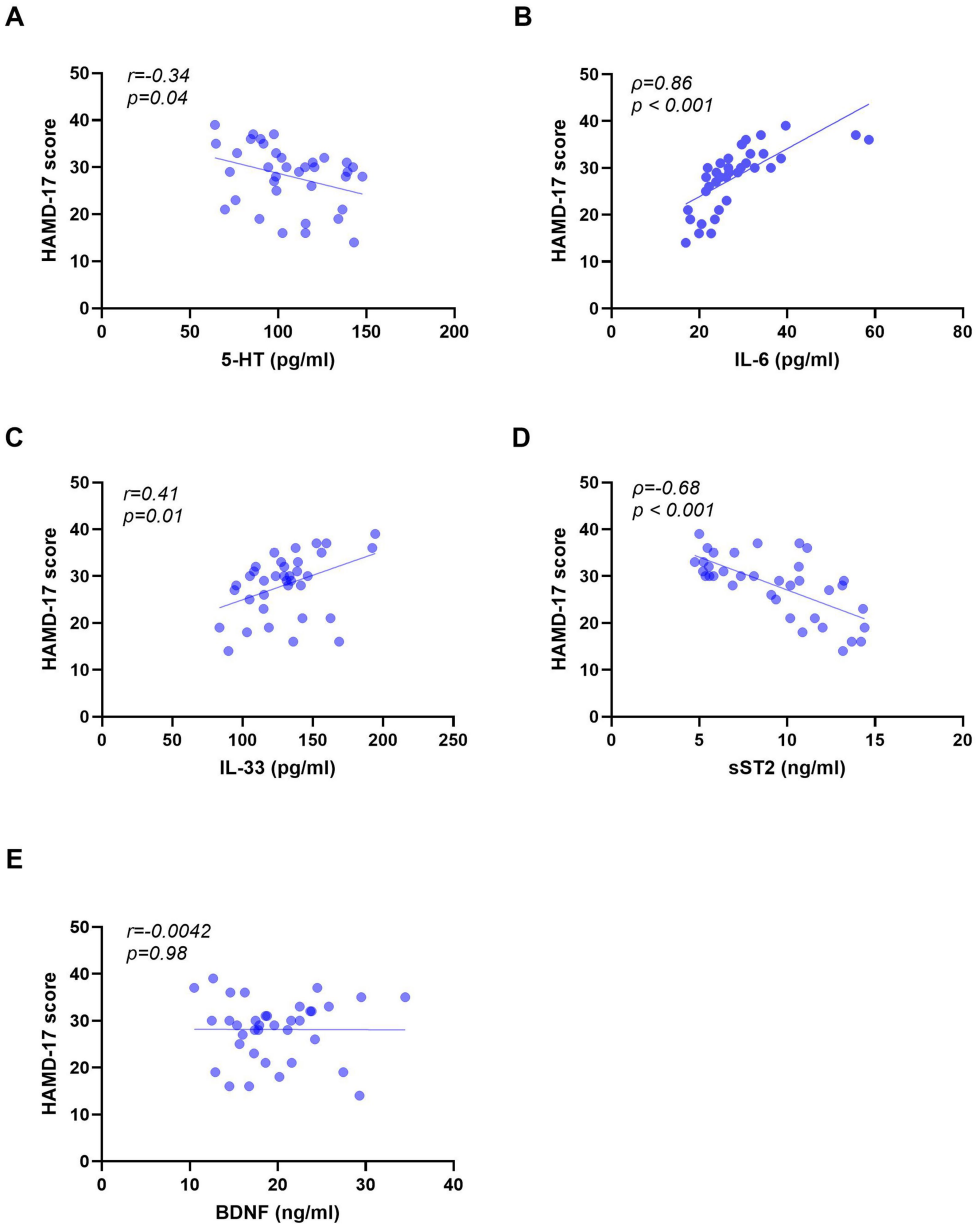
Correlations between baseline serum biomarker levels and baseline depression severity. Scatter plots showing the relationship between serum levels of **(A)** 5-HT, **(B)** IL-6, **(C)** IL-33, **(D)** sST2, and **(E)** BDNF and the 17-item Hamilton Depression Rating Scale (HAMD-17) score before rTMS treatment. The correlation coefficient (r) and corresponding *p*-value are displayed in each panel. Normality was assessed using the Shapiro–Wilk test. Pearson correlation coefficients (r) are reported for normally distributed biomarkers (5-HT, IL-33, BDNF), whereas Spearman’s rank correlation coefficients (*ρ*) are reported for non-normally distributed biomarkers (IL-6, sST2). Nominal *p*-values are displayed in each panel. All *p*-values were adjusted for multiple comparisons using the FDR procedure. Statistical significance after FDR correction is indicated by asterisks in the respective panels.

To explore the relationship between the dynamics of peripheral biomarkers and the severity of depression, we compared the levels of 5-HT, IL-6, IL-33, sST2, and BDNF in the serum of all patients before the rTMS treatment course with their baseline HAMD-17 scores. The results showed that the serum IL-6 level was significantly positively correlated with the HAMD-17 score (Spearman *ρ* = 0.86, nominal *p <* 0.001; FDR corrected *q <* 0.001; Figure 1B). The sST2 level was significantly negatively correlated with the HAMD-17 score (Spearman *ρ* = −0.68, nominal *p <* 0.001; FDR corrected *q <* 0.001; Figure 1D). The serum IL-33 level was also significantly positively correlated with the HAMD-17 score (Pearson r = 0.41, nominal *p* = 0.010; FDR corrected *q =* 0.017; Figure 1C). The 5-HT level showed a nominal negative correlation with the HAMD-17 score (Pearson r = −0.34, nominal *p* = 0.043), but this association did not pass the multiple comparison correction (FDR corrected *q =* 0.054; Figure 1A). There was no significant correlation between the BDNF level and the HAMD-17 score (Pearson r = −0.004, *p* = 0.98; Figure 1E).

### 3.3 Changes in biomarker levels after rTMS treatment

**Figure 2:**
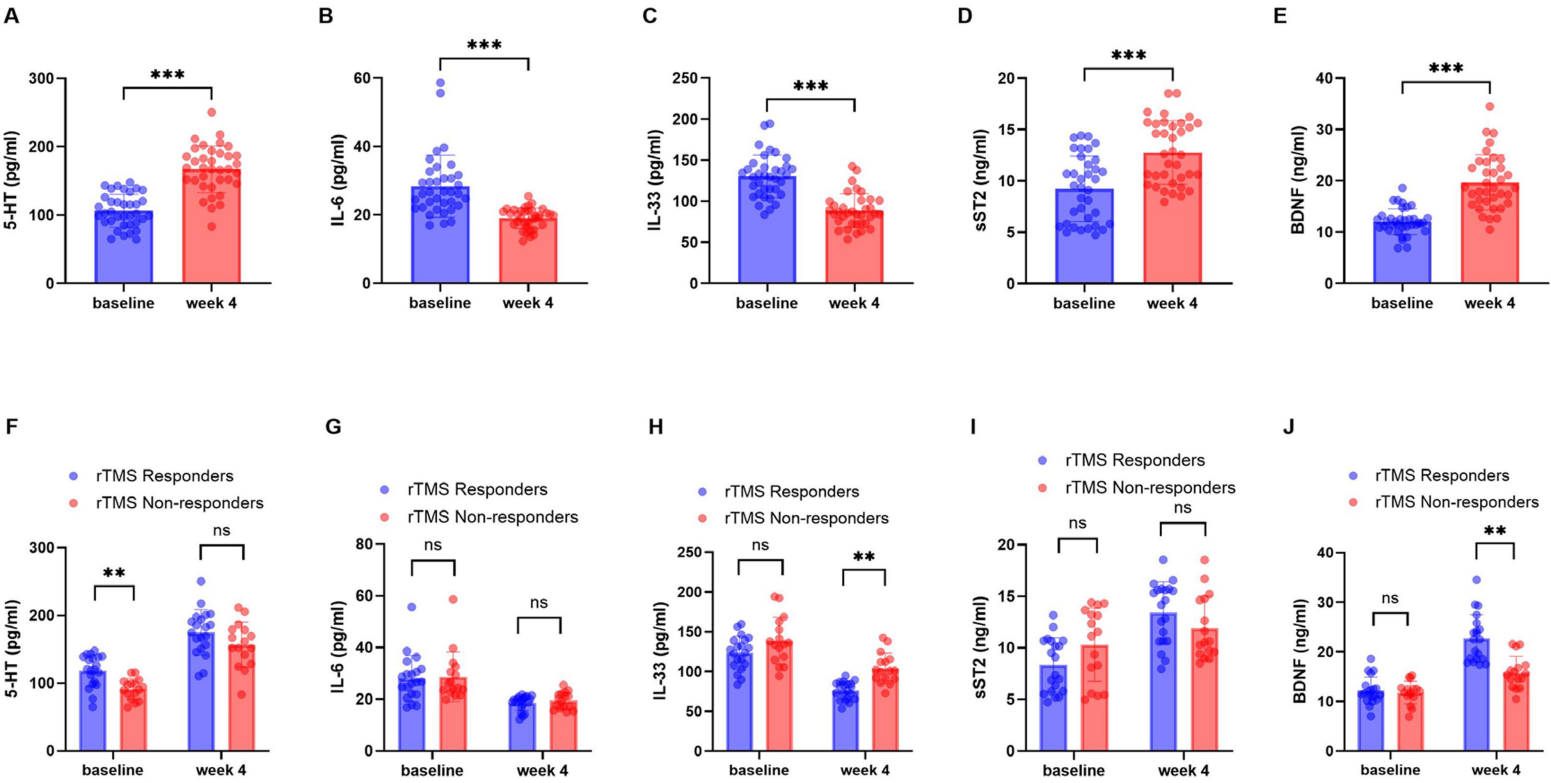
Changes in serum biomarker levels following rTMS treatment and their association with clinical response. **(A-E)** Comparison of serum biomarker levels before (Baseline) and after (Week 4) rTMS treatment in the entire cohort (*n =* 36) for **(A)** 5-HT, **(B)** IL-6, **(C)** IL-33, **(D)** sST2, and **(E)** BDNF. **(F-J)** Comparison of biomarker levels between Responders (R, *n =* 20) and Non-responders (NR, *n =* 16) after 4 weeks of rTMS treatment for **(F)** 5-HT, **(G)** IL-6, **(H)** IL-33, **(I)** sST2, and **(J)** BDNF. All *p*-values were adjusted for multiple comparisons using the FDR procedure. Statistical significance after FDR correction is indicated by asterisks in the respective panels (*q < 0.05, **q < 0.01, ***q < 0.001).

We measured the serum levels of IL-33, sST2, 5-HT, IL-6, and BDNF in patients before and after receiving rTMS treatment. In the overall patient group, the levels of serum IL-33 and IL-6 significantly decreased after treatment (nominal *p <* 0.001; FDR corrected *q <* 0.001; Figures 2B,C), while the levels of 5-HT, sST2 and BDNF significantly increased (nominal *p <* 0.001; FDR corrected *q <* 0.001; Figures 2A,D–E).

Further comparisons were made between the clinical responders and non-responders regarding the differences in these indicators (Figures 2F–J). The results showed that the baseline 5-HT level was significantly higher in responders than in non-responders (nominal *p <* 0.001; FDR corrected *q =* 0.010; Figure 2F). At the end of treatment, the serum IL-33 level of responders was significantly lower than that of non-responders (nominal *p* = 0.002; FDR corrected *q =* 0.007; Figure 2H), while the BDNF level was significantly higher than that of non-responders (nominal *p <* 0.001; FDR corrected *q =* 0.005; Figure 2J). There were no significant differences in the levels of 5-HT, IL-6 and sST2 at the other time points between the two groups (all FDR corrected *q* > 0.05).

### 3.4 Association between biomarker change values and clinical response and symptom improvement

**Figure 3:**
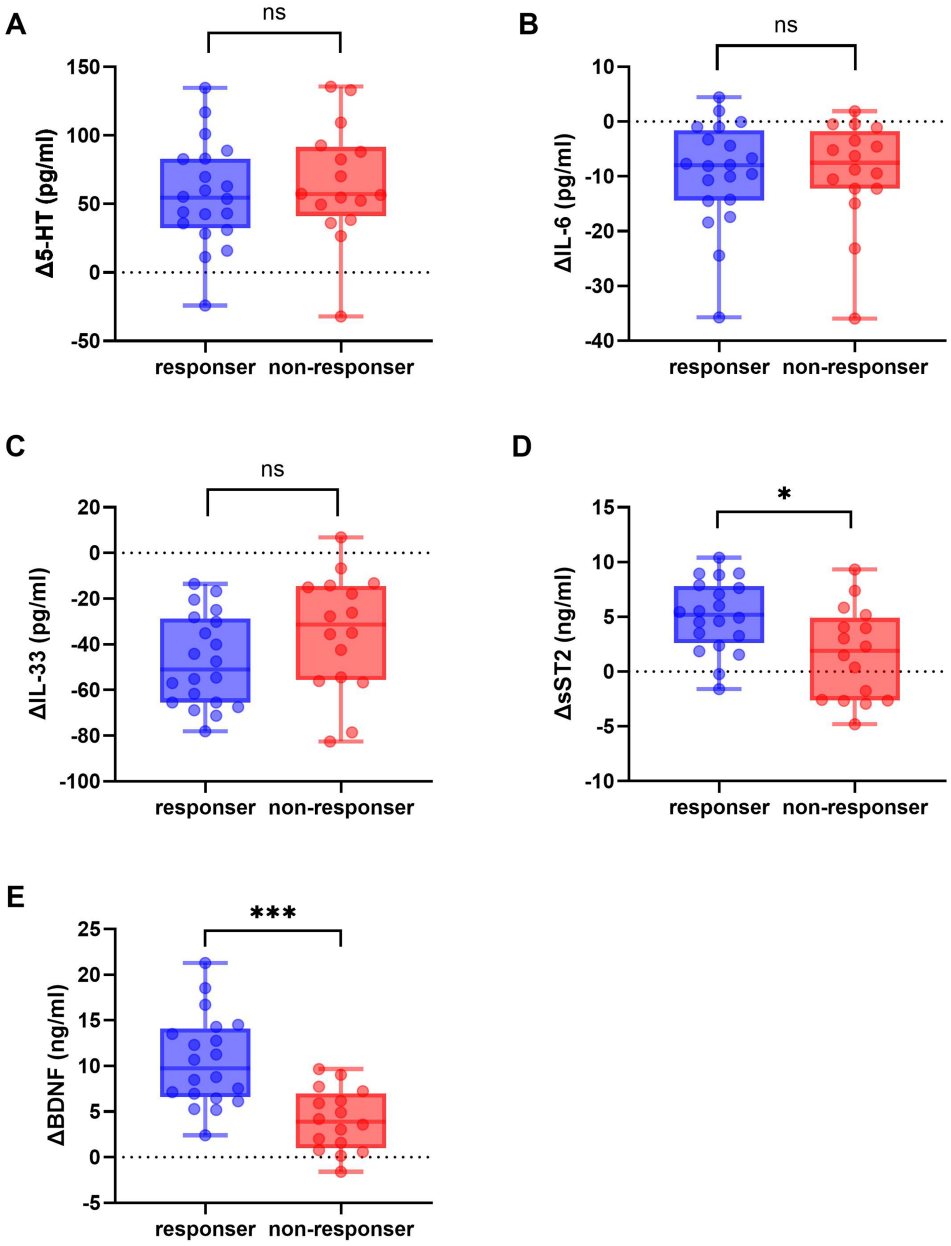
Comparison of pre-to-post changes in serum biomarker levels between rTMS responders and non-responders. Box-and-whisker plots with superimposed individual data points showing the change (*Δ* = week 4 − baseline) in serum levels of **(A)** 5-HT, **(B)** IL-6, **(C)** IL-33, **(D)** sST2, and **(E)** BDNF. Normality of Δ values was assessed using the Shapiro–Wilk test. Independent-samples *t*-tests were used for normally distributed Δ values (5-HT, IL-33, sST2, BDNF), and Mann–Whitney U tests were used for non-normally distributed Δ values (IL-6). To account for multiple testing across the five biomarkers, all *p-values* were adjusted using the Benjamini-Hochberg false discovery rate (FDR) procedure. Asterisks denote between-group differences that remained significant after FDR correction (*q < 0.05, ***q < 0.001).

To further evaluate the clinical significance of the dynamic changes in biomarkers, we calculated the change values (*Δ* = 4 week - baseline) before and after treatment for each indicator, and compared the differences between the response group and the non-response group (Figure 3). After FDR multiple comparison correction, the increase amplitude of ΔBDNF in the response group was significantly greater than that in the non-response group (nominal *p* = 0.00009, corrected *q =* 0.0004; Figure 3E). The increase amplitude of ΔsST2 in the response group was also significantly greater than that in the non-response group (nominal *p* = 0.0084, corrected *q =* 0.021; Figure 3D). The change values of the other indicators showed no significant differences between the two groups (all corrected *q* > 0.05; Figures 3A–C).

**Figure 4:**
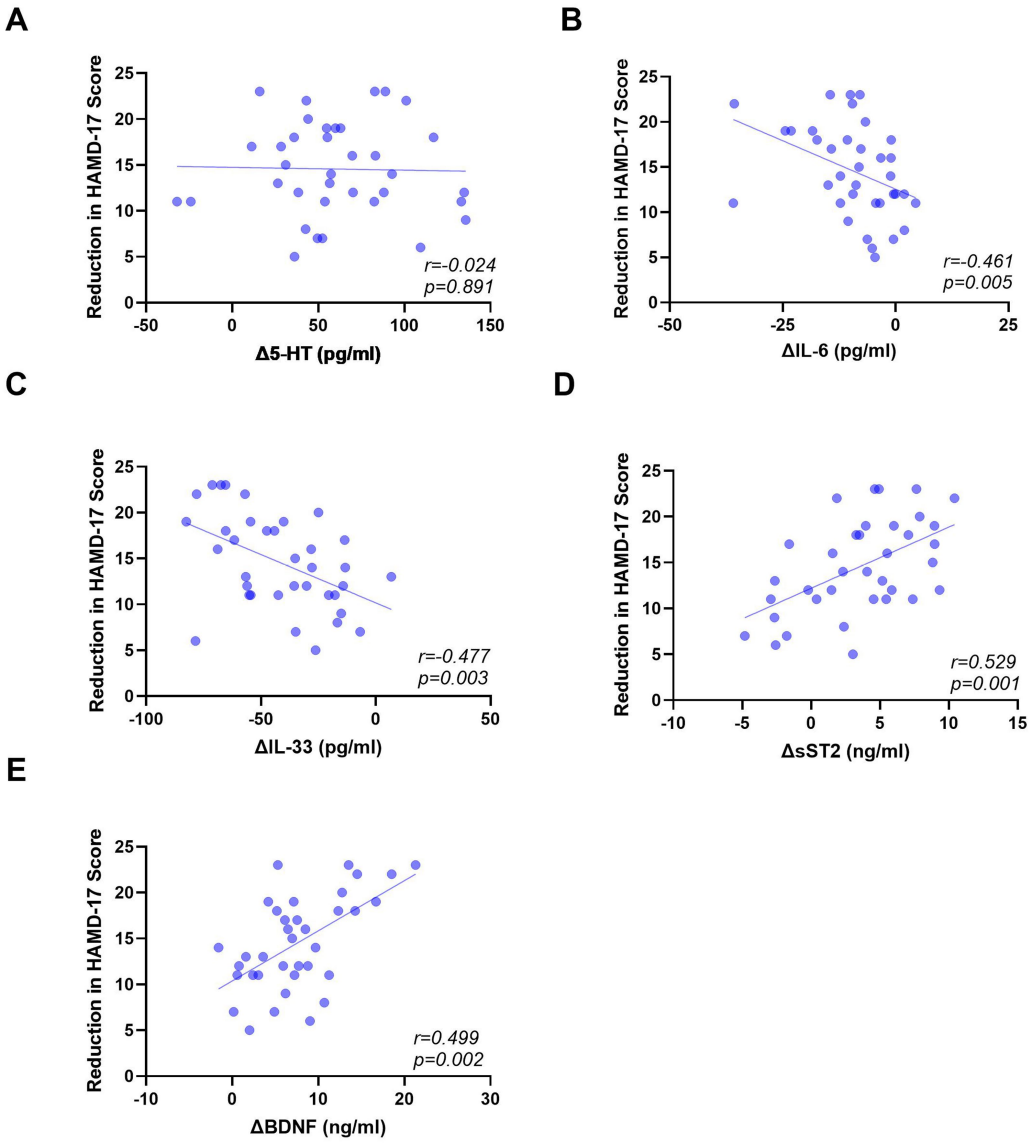
Correlations between changes in serum biomarker levels and improvement in depressive symptoms. Scatter plots illustrating the relationship between pre-to-post changes in serum biomarker levels (Δ = Week 4 − Baseline), including **(A)** 5-HT, **(B)** IL-6, **(C)** IL-33, **(D)** sST2, and **(E)** BDNF, and the reduction in 17-item Hamilton Depression Rating Scale scores (ΔHAMD = Baseline − Week 4). Positive ΔHAMD values denote greater symptomatic improvement. Pearson correlation coefficients (r) are reported for normally distributed biomarkers (5-HT, IL-33, sST2, and BDNF); a Spearman rank correlation coefficient (*ρ*) is reported for IL-6 due to non-normal distribution. Nominal *p*-values are displayed in each panel. To account for multiple testing across the five biomarkers, *p*-values were adjusted using the Benjamini-Hochberg False Discovery Rate (FDR) procedure.

In addition, we analyzed the correlation between biomarker change values and the degree of depression symptom improvement (ΔHAMD = baseline - 4 week) (Figure 4). After FDR correction, ΔIL-6 (Spearman *ρ* = −0.46, corrected *q =* 0.0058), ΔIL-33 (Pearson r = −0.48, corrected *q =* 0.0054) were significantly negatively correlated with ΔHAMD, and ΔsST2 (Pearson r = 0.53, corrected *q =* 0.0045) and ΔBDNF (Pearson r = 0.50, corrected *q =* 0.0049) were significantly positively correlated with ΔHAMD. Δ5-HT showed no significant correlation with ΔHAMD (Pearson r = 0.02, corrected *q =* 0.891; Figure 4A).

### 3.5 Association of biomarkers with rTMS response and their discriminatory performance

**Figure 5:**
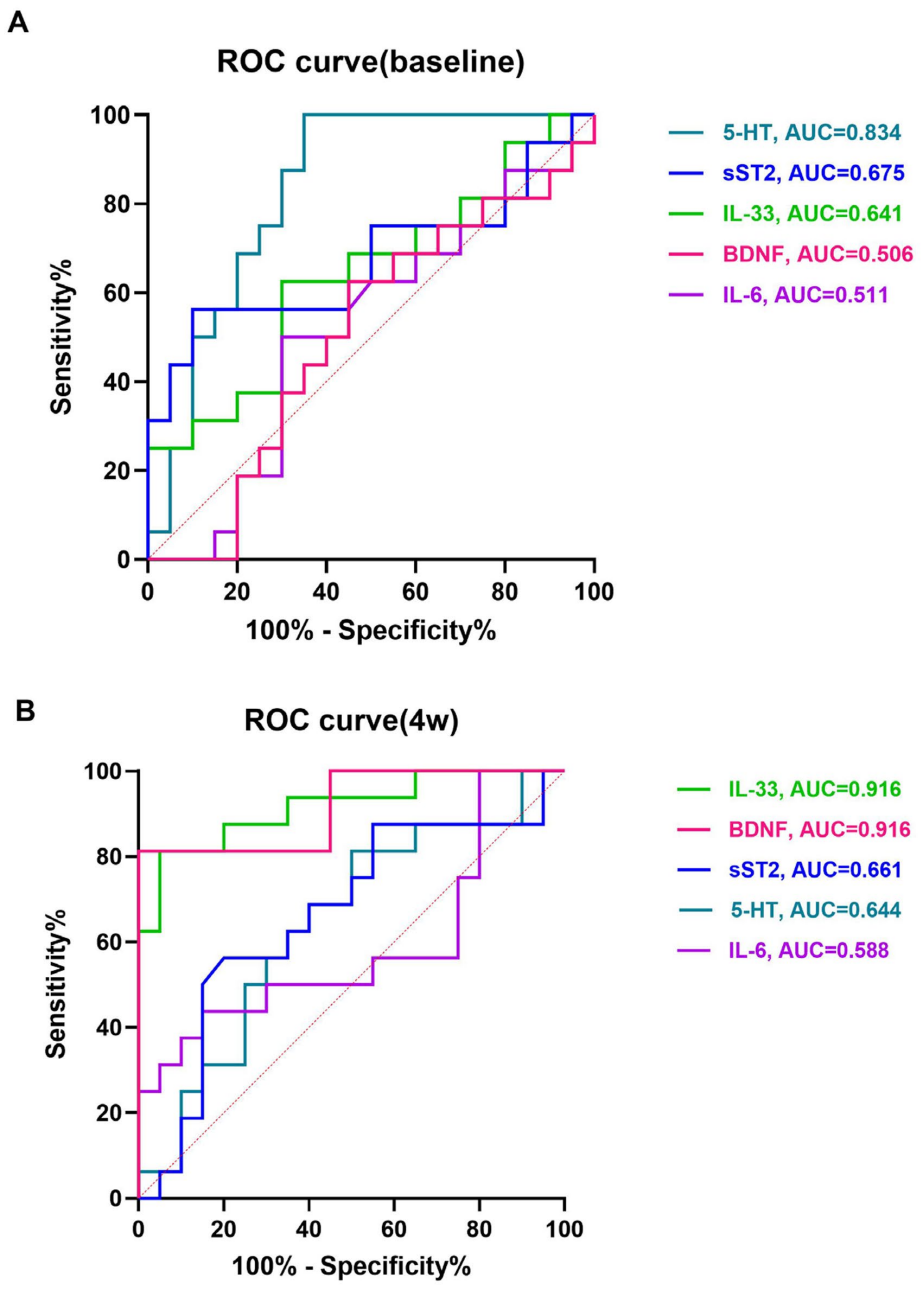
Receiver operating characteristic (ROC) curves for the association of serum biomarkers with rTMS treatment response. **(A)** ROC curves for baseline serum levels of 5-HT, sST2, IL-33, BDNF, and IL-6 in predicting treatment response after 4 weeks of rTMS. **(B)** ROC curves for serum levels of the same biomarkers measured at week 4 in predicting treatment response. The AUC for each biomarker is calculated.

To evaluate the potential of the measured biomarkers as predictive tools, we conducted ROC curve analysis (Figure 5) and calculated the AUC, confidence intervals, significance and the optimal cut-off value (based on the Youden index) (Table 2). The analysis included five key indicators before treatment and after 4 weeks of treatment: 5-HT, IL-6, IL-33, sST2, and BDNF. Prior to rTMS treatment, the serum 5-HT level could predict the treatment response, with an AUC of 0.834 (95% CI: 0.700–0.969, *p <* 0.001), and the optimal cut-off value was 115.56 pg./mL. In contrast, sST2, IL-33, IL-6, and BDNF did not significantly predict response (all AUCs close to 0.5, *p* > 0.05). After 4 weeks of treatment, the serum IL-33 and BDNF levels showed strong discriminatory ability in differentiating responders from non-responders, with AUCs of 0.916 (IL-33 95% CI: 0.819–1.000; BDNF 0.818–1.000, both *p <* 0.001). The optimal cut-off value for IL-33 was 90.16 pg./mL, and for BDNF it was 17.36 ng/mL. In contrast, the 4-week levels of IL-6 and sST2 showed no significant discriminatory ability (both *p* > 0.05).

**Table 2:** Results from ROC curve analysis.

Characteristic	AUC (95% CI)	*P-*value	Cut off	Sensitivity (%)	Specificity (%)	Youden index
5-HT-baseline	0.834 (0.700–0.969)	<0.001	115.56	100.00	65.00	0.65
5-HT-4w	0.644 (0.459–0.828)	0.140	–	–	–	–
IL-6-baseline	0.511 (0.317–0.705)	0.910	–	–	–	–
IL-6-4w	0.588 (0.387–0.788)	0.370	–	–	–	–
IL-33-baseline	0.641 (0.455–0.827)	0.150	–	–	–	–
IL-33 -4w	0.916 (0.819–1.000)	<0.001	90.16	81.25	95.00	0.76
sST2-baseline	0.675 (0.484–0.867)	0.070	–	–	–	–
sST2-4w	0.661 (0.474–0.848)	0.100	–	–	–	–
BDNF-baseline	0.506 (0.312–0.700)	0.950	–	–	–	–
BDNF-4w	0.916 (0.818–1.000)	<0.001	17.36	81.25	100.00	0.81

AUC, area under the receiver operating characteristic curve; CI, confidence interval. Cut-off values were determined by maximizing Youden’s index (sensitivity + specificity − 1). For biomarkers with AUC not significantly different from 0.5 (*p* > 0.05), cut-off values and diagnostic indices are not reported. Units: 5-HT and IL-33 in pg/ml; BDNF in ng/ml.

## 4 Discussion

This study investigated the changes of serum IL-33, sST2 signaling pathway and other related biomarkers (5-HT, IL-6, and BDNF) during rTMS treatment for MDD. Our main findings are as follows: At baseline, the inflammatory markers IL-33 and IL-6 levels were positively correlated with depression severity, while sST2 and 5-HT levels showed negative correlations. After 4 weeks of rTMS treatment, responders exhibited a significant decrease in peripheral blood IL-33 levels and a significant increase in BDNF levels. The degree of increase in BDNF and the degree of decrease in IL-33 were both significantly correlated with the individual improvement in depressive symptoms (ΔHAMD). The baseline 5-HT level had predictive value for treatment response, and the levels of IL-33 and BDNF after treatment showed a stronger correlation with treatment response (AUCs were both 0.916), reflecting their potential as efficacy correlation markers. These results suggest that the antidepressant efficacy of rTMS may be partially achieved by modulating the balance between neuroimmune and neurotrophic factors, and provide potential directions for biomarker-based efficacy prediction.

IL-33 is a pleiotropic cytokine that is abundantly expressed in the CNS, primarily found in astrocytes, with lower expression in oligodendrocytes, microglia, and neurons (Vainchtein et al., 2018; Sun et al., 2021). IL-33 is released by damaged cells as an “alarmin,” acting through its receptor ST2 and the IL-1 receptor family ligand (IL-1RAcp) (Fonta et al., 2025). Clinical studies have shown that depression leads to a significant increase in the serum pro-inflammatory cytokine IL-33, along with elevated oxidative stress levels (HO-1, iNOS) (Ogłodek and Just, 2018). A clinical study involving 160 stroke patients revealed that serum IL-33 and hsCRP levels were significantly higher, while sST2 levels were significantly lower in the post-stroke depression group compared to the non-depression group (Xu and Wu, 2021). IL-33 was positively correlated with depression quantitative scores, whereas sST2 was negatively correlated with these scores (Xu and Wu, 2021). We found that in untreated MDD patients, serum IL-33 levels showed a significant positive correlation with the severity of depressive symptoms (r = 0.41), a result consistent with the findings from the aforementioned post-stroke depression study. Another clinical study indicated that an IL-33 concentration ≤71.85 ng/L is an independent predictor of post-stroke depression (95% CI: 1.129–7.515, *p* = 0.027) and a protective prognostic factor for patients with acute ischemic stroke (95% CI: 0.954–0.997, *p* = 0.024) (Chen et al., 2020). Our study demonstrated consistency, with post-treatment serum IL-33 levels achieving an AUC of 0.916 for rTMS treatment response in MDD patients. This suggests that IL-33 is not only a serum marker associated with depression severity but also holds promise as a predictive indicator for treatment response.

Several studies have investigated the mechanisms by which IL-33 influences depression. For example, transcriptomic analysis revealed that in the hippocampi of IL-33-overexpressing mice, pathways related to metabolism, immunity, and circadian rhythms were significantly altered, with mitochondrial dynamics and surveillance pathways being the most enriched (Li et al., 2025). The study found that after IL-33 was released as an “alarmin,” it inhibited BDNF expression through the NF-κB pathway, thereby suppressing GABAergic transmission in the amygdala, while NF-κB inhibitors abolished the effects of IL-33 on depression (Zhuang et al., 2022). Gao et al. also confirmed in the Chronic Unpredictable Mild Stress (CUMS) mouse model that the IL-33/ST2-MyD88 pro-inflammatory pathway was significantly activated in the serum and hippocampus of depressed mice, and effective intervention could down-regulate this pathway and alleviate depressive-like behaviors (Gao et al., 2025). In this study, a significant decrease in IL-33 levels was observed in responders to rTMS, and the decrease was negatively correlated with the degree of symptom improvement (r = −0.58, *p <* 0.001), accompanied by a significant increase in BDNF levels. This suggests that the antidepressant efficacy of rTMS may be related to the reduction of biomarkers in the IL-33/ST2 pathway, while regulating the expression of BDNF, thereby promoting neural repair.

In addition to the aforementioned classical pro-inflammatory pathways, IL-33 also demonstrates significant neuroprotective and repair potential. Under non-inflammatory or acute injury conditions, IL-33 can promote the expression of M2-type macrophages, mediate the inhibition of inflammatory responses through the NF-κB pathway, and participate in tissue homeostasis, development, and remodeling (Molofsky et al., 2015). At the same time, IL-33 can exert anti-inflammatory functions by regulating immune cells. For example, the expression of IL-33 receptor ST2 is severely upregulated in Treg cells, and IL-33 can regulate the differentiation and function of T cells (Tregs), promote the production of anti-inflammatory cytokines (such as IL-10), thereby inhibiting the expression of pro-inflammatory cytokines and actively suppressing the persistence and expansion of inflammatory responses (Siede et al., 2016). A meta-analysis result shows that the levels of serum and cerebrospinal fluid IL-33 and sST2 are positively correlated with the reduced risk of depression (Liu et al., 2023). This difference may be due to the differences in whether the study population already has depression, and it also indicates the complex dual role of IL-33 in depression and other diseases.

It is worth noting that this study found that the level of sST2 significantly increased after rTMS treatment, and the increase in the response group was significantly higher than that in the non-response group. sST2 acts as an agonistic receptor to competitively bind to IL-33, blocking the activation of downstream signals. Therefore, rTMS may not simply inhibit IL-33, but rather by upregulating sST2, it converts the signal direction of IL-33, reducing the inflammatory effect while retaining its neuroprotective effect. This is in line with the results of responders showing a decrease in IL-33 and an increase in BDNF, suggesting that the efficacy of rTMS is partly due to the regulation of the signal balance of the IL-33/ST2 axis, rather than simple inhibition of inflammation. Currently, clinical trials related to IL-33 mainly focus on regulating its receptor ST2 and inhibiting the IL-33 signaling pathway, and play a key role in various inflammatory diseases such as depression. However, the dual regulatory mechanism of IL-33 on neuroinflammation in MDD has not been fully determined. The interaction between IL-33 and other inflammatory cytokines has not been thoroughly explored, and given its role in a complex pathological environment, it remains an urgent issue. Future research should further explore the cell-specific effects of IL-33 in different disease stages and different brain regions.

Both 5-HT and BDNF play important roles in MDD. Evidence shows that the levels of BDNF and 5-HT in the serum of patients with depression are decreased, and effective antidepressant treatment can reverse this change (Esalatmanesh et al., 2023). Clinical studies have shown that rTMS treatment can affect the levels of BDNF and 5-HT in the bodies of depressed patients. For example, using rTMS combined with physical exercise can significantly reduce the depressive mood of patients with methamphetamine use disorder and accompanied by a significant increase in 5-HT in the blood (Wang et al., 2026). For 5-HT, this study verified that the level of 5-HT is negatively correlated with the severity of depression, and also revealed its unique predictive value. MDD patients with higher baseline 5-HT levels showed better responses to rTMS treatment (AUC = 0.834). However, this predictive advantage disappeared after treatment, and there was no significant difference in the change amount of 5-HT before and after treatment between the response group and the non-response group, and the change amplitude was also not significantly correlated with the degree of symptom improvement. These findings collectively suggest that the clinical efficacy mechanism of rTMS may not mainly rely on the direct and continuous regulation of 5-HT levels.

This study also found that, 4 weeks after rTMS treatment, the level of BDNF in the patients’ serum significantly increased. After the treatment ended, the content of serum BDNF in the treatment response group was significantly higher than that in the non-response group (FDR correction *q =* 0.005). Further analysis indicated that the increase in BDNF in the response group was significantly greater than that in the non-response group, and the increase in BDNF was significantly positively correlated with the improvement of depressive symptoms. The level of BDNF after treatment became an indicator for predicting efficacy (AUC = 0.916). The above results cross-validated the key role of BDNF in the antidepressant effect of rTMS from multiple dimensions. In the inflammatory pathway, this study found that the level of IL 33 decreased overall after rTMS treatment, and the degree of decrease in IL 33 was significantly negatively correlated with symptom improvement. Similarly, sST2 increased overall after treatment, and the increase in the response group was significantly greater than that in the non-response group, and its increase degree was also positively correlated with symptom improvement. Combining the results related to BDNF and IL 33, it was found that there might be a functional antagonistic relationship between IL-33 and BDNF. The antidepressant effect of rTMS may contain a dual mechanism, ranging from a decrease in IL-33/ST2-related inflammatory markers to an increase in BDNF levels.

This study has several limitations. Firstly, this study is a preliminary exploratory study and no prior sample size estimation was conducted. The sample size is relatively small (*n =* 36) and the small sample size increases the risk of Type I errors, especially for subgroup analysis and ROC curve analysis. The high AUC values (both 0.916) for post-treatment IL-33 and BDNF should be interpreted with caution. Due to the small sample size, ROC analyses are susceptible to overfitting, meaning the reported AUCs may be overoptimistic and sample-specific. Therefore, the high predictive efficacy of IL-33 and BDNF presented in this study requires external validation in an independent, large-sample prospective cohort. Secondly, this study did not have a control group (such as a healthy control group or a sham-stimulation control group). Hence, it would be difficult to determine whether the observed changes in biomarker levels (such as the decrease in IL-33 and the increase in BDNF) are specifically attributed to rTMS treatment or may be influenced by natural disease course, drug effects, or non-specific factors. Moreover, due to the lack of a control group, we cannot determine whether the levels of biomarkers after treatment return to the “normal” range. Future research should include healthy controls or a randomized sham-controlled design to more clearly evaluate the regulatory effects of rTMS on the IL-33/ST2 pathway and BDNF. Thirdly, this study is an observational design, which has a significant limitation in explaining the causal relationship between the changes in IL-33/ST2 and the efficacy of rTMS. Future research can further clarify the mechanism by combining animal models or intervention experiments. Fourthly, the patients enrolled in this study were limited to the age range of 18–65 years, excluding elderly patients over 65 years old. rTMS has a good clinical application prospect due to its small side effects and the ability to reduce the risk of polypharmacy. Multiple studies have shown that rTMS has a definite therapeutic effect on elderly depression (typically defined as ≥60 years old) (Valiengo et al., 2022; Almheiri et al., 2023). Future research should specifically include elderly depression cohorts to verify the findings of this study. In addition, this study allowed patients to receive rTMS treatment on the basis of stable doses of antidepressants, but it cannot completely rule out the potential influence of drugs on the levels of serum 5-HT, BDNF, etc. The sample size of this study was limited, and no stratified analysis or statistical control was performed for drug types. Future research can consider including patients who have not taken medication or using a washout period design to more purely evaluate the independent effect of rTMS.

## 5 Conclusion

In this prospective cohort study involving 36 MDD patients, effective rTMS treatment was accompanied by synergistic changes in serum biomarkers, with reductions in IL-33 and IL-6 levels and increases in BDNF and sST2 levels. Of note, the degree of increase in BDNF and decrease in IL-33 were significantly correlated with the magnitude of individual symptom improvement, suggesting an association between neuroimmune and neurotrophic modulation and clinical recovery. The above findings suggest that IL-33/ST2 and BDNF pathways may be jointly involved in the antidepressant mechanism of rTMS, and the trajectories of these biomarkers are expected to be used to monitor treatment response. Given the limited sample size, future larger studies are needed to validate these findings and establish their clinical utility.

## Data Availability

The original contributions presented in the study are included in the article/supplementary material, further inquiries can be directed to the corresponding author.
